# Reconstruction of Metacarpals of Two Rays with Double Barrel Osteocutaneous Fibular Flap in a Hand Injury with Composite Tissue Loss: A Case Report

**DOI:** 10.5704/MOJ.2411.010

**Published:** 2024-11

**Authors:** JK Mishra, SA Sahu, A Sindhuja, BK Kar, A Saha

**Affiliations:** 1 Department of Burns and Plastic Surgery, All India Institute of Medical Sciences, Raipur, India; 2 Department of Orthopaedics, All India Institute of Medical Sciences, Raipur, India

**Keywords:** metacarpal reconstruction, free fibula flap, metacarpal loss

## Abstract

Free fibula flap has been a workhorse for head, neck, and extremity long bone defects. We discuss the reconstruction challenge in an unusual hand injury case involving the loss of multiple metacarpals and soft tissue with surprising preservation of finger vascularity. The reconstructive goals were addressed with a microvascular osteocutaneous fibula flap transfer with multiple osteotomies to create spitting images of metacarpals and soft tissue defects restored with the skin paddle. The outcome, in terms of functional gain, was sufficient for managing day-to-day activities. We share our experience in reconstructing this unique presentation of a complex hand injury.

## Introduction

Occupational hazards are one of the common causes of hand injuries, with fingers (67.5%) being the most involved, followed by palm and dorsum^1^. Sometimes, hand injuries are complicated by composite tissue loss, including bone, soft tissue, tendons, and joints. The loss of metacarpals with intact finger vascularity due to injury is uncommon. It results in the absence of a leverage system of the viable distal fingers, which become non-functional. Various options for metacarpal reconstruction include the fibula flap, scapula flap, medial femoral condyle flap, and serratus anterior rib composite flap^2^. However, the fibula flap is most suitable for metacarpal reconstructions due to its inherent properties matching those of the metacarpals.

**Fig. 1:**
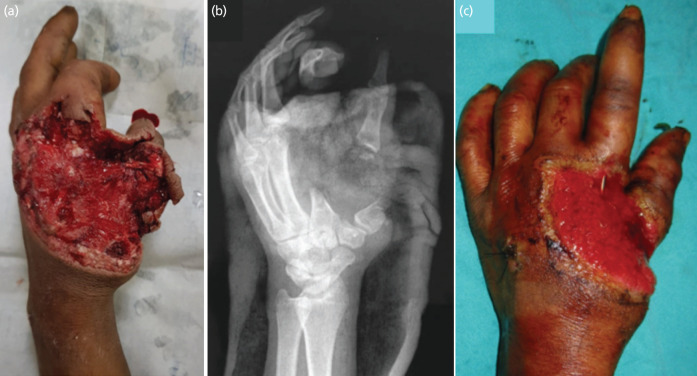
(a) Image of the injured hand following trauma showing composite tissue loss. (b) Radiograph of the injured hand showing loss of 1st and 2nd metacarpals. (c) Clinical image showing stabilisation of 1st and 2nd digits with K-wire.

We present a complex hand injury reconstruction case with loss of 1st and 2nd metacarpals and overlying soft tissue with viable distal fingers using a free osteocutaneous fibula flap (Fig. 1a and b). Following reconstruction, the patient experienced a satisfactory functional outcome.

## Case Report

A 17-year-old male injured his left hand with a power press machine. He presented with composite tissue loss involving first and second metacarpals, metacarpophalangeal joints, extensor tendons of the corresponding digits, and soft tissue over the first web and dorsum. The associated soft tissue defect was about 10 x 8cm (Fig. 1a). Although the left thumb and index finger vascularity remained intact, their function was compromised. The sensation and function of long flexor tendons were intact in the index finger and thumb. However, the patient was not able to do the flexion due to the loss of the bony lever.

The capillary refilling time was normal in both digits. Emergency reconstruction was deferred due to contamination. Initially, wound toileting and k-wire fixation of the first and second digits to the remaining stump of metacarpals and carpals was done temporarily to prevent hanging/rotation of the distal fingers, avoiding spasm of digital arteries and further damage (Fig 1c). Elective reconstruction was planned two weeks following the injury.

Reconstruction technique: the apparent bone gap was calculated with k-wires in situ. The fibula bone size was taken 1cm in excess for each metacarpal, anticipating the bone defect might increase after k-wire removal. The first metacarpal gap was reconstructed using the proximal 4cm of the fibula bone, while the second metacarpal bone gap was reconstructed by a distal 5cm of the fibula bone. To maintain the web space, the intervening 8cm of fibula bone was filleted, preserving the continuity of the peroneal pedicle in the periosteal sleeve for perfusion of the distal fibula segment.

**Fig. 2:**
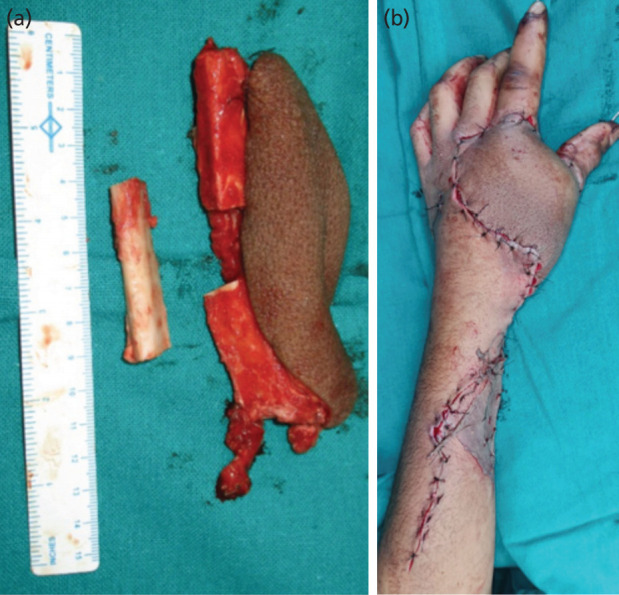
(a) Harvested Osteocutaneous fibula flap with filleted middle segment. (b) Reconstructed hand after flap inset.

A skin paddle (12x10cm) supplied by a perforator attached to the proximal bone segment was designed to reconstruct the soft tissue defect of the dorsum and first web space (Fig. 2). Any kinking or compression of the perforators supplying the skin paddle was carefully avoided while fixing the fibula bone segments with k-wires. As it was an open contaminated wound and there were chances of infection, k-wire fixation rather than plate fixation was done as the plate may require removal later. The post-operative period was uneventful, and k-wires were removed after four weeks of reconstruction.

**Fig. 3:**
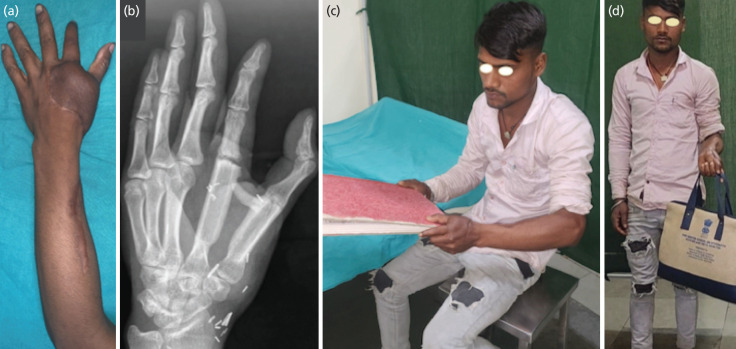
(a) Follow-up image showing a well-settled flap. (b) Follow-up radiograph showing fibular bone segments resembling metacarpals without bony resorption after six months. (c) Patient holding a book (Power grasp). (d) Patient lifting a bag (hook grip).

There was no bony resorption of the reconstructed metacarpals observed on the radiograph with durable soft tissue cover on six months follow-up (Fig. 3b). The patient can perform his routine activities, although extension of the first and second fingers was not possible. Pinch grip was absent during follow-up as the patient is unable to flex the DIP joint of the index finger. However, the power grasp (Grade 4) and hook grip (Grade 4) are present due to adduction movement of the thumb at the CMC joint and 0-30 active flexion at the DIP joint (Fig. 3c and 3d). The Disabilities of the Arm, Shoulder, and Hand (DASH) score of the reconstructed hand is 39 correlating to moderate disability.

## Discussion

Reconstruction of the post-traumatic hand defect with composite tissue loss is challenging. The primary goal of reconstructive surgery for a traumatized hand should be to achieve a pain-free limb that is aesthetically acceptable and capable of a good range of movement for performing day-today tasks. The choice of reconstruction method for metacarpal defects depends on several factors, including the status of the remaining bone segment, distal finger vascularity, associated soft tissue loss, and any injury to major neurovascular structures, joints, and tendons. Traumatic segmental defects of the metacarpals with intact distal fingers are uncommon scenarios. There are few reported cases of such a reconstruction by a vascularised osteocutaneous fibula flap.

In 1975, Taylor described the microvascular transfer of the fibula flap for the first time, primarily for tibia reconstruction^3^. Since then, it has become the gold standard for extremity long bone reconstruction^4^. However, the use of vascularised fibula for multiple metacarpal reconstructions is still not usually done, and there is a need for more literature on this topic. The fibula, a straight cortical bone, provides a good size match for metacarpals. Due to the rich periosteal blood supply, osteotomies can be performed at desired lengths to reconstruct multiple metacarpals. The vascularised graft provides better wound healing and a lower infection rate than corticocancellous allografts^5^. Additionally, osseous integration is much better with minimal or no bone resorption in long-term follow-up compared to avascular bone grafts. The medullary cavity of the fibular flap provides restraint placement for the prosthesis; hence MCP joint reconstruction can be carried out^5^. Functional outcomes are better with additional procedures. As the index finger and thumb are the important fingers of the hand, attributing to the significant functional aspect, preservation of these fingers is of prime preference while dealing with hand trauma. Depending on the mode of injury and the degree of contamination, the reconstruction can be planned in an emergency or elective manner.

In composite defects of the hand with contamination, with a scenario like us, where multiple metacarpals are lost but distal vascularity is preserved, unsupported fingers can be temporarily stabilized with k-wires in an emergency until elective reconstruction is planned with a vascularized bone flap a few days later. During this period, the wound is cleared of contamination and oedema. Elective reconstruction with a free fibula flap restores the bony axis of the fingers, salvaging them. In our case, reconstruction of the extensor tendons was planned at later stage, after attaining durable tissue cover but patient was not willing to undergo further corrective surgical procedure. By preserving the first and second rays, the patient was able to resume daily activities and return to work at a wood fabrication factory, performing the same task.

Proper planning with multiple surgeries is required in a mangled injury case for a good functional outcome. Many options are available for the reconstruction of metacarpals, but composite reconstruction of multiple metacarpals with overlying soft tissue defects presents a significant challenge. The free osteocutaneous fibula is optimal for reconstructing multiple metacarpals with soft tissue defects in traumatic hand injuries. Post-operatively, the patient achieves good functional outcomes with minimal rehabilitation time.
